# Retrospective assessment of at-risk myocardium in reperfused acute myocardial infarction patients using contrast‐enhanced balanced steady‐state free‐precession cardiovascular magnetic resonance at 3T with SPECT validation

**DOI:** 10.1186/s12968-021-00730-7

**Published:** 2021-03-15

**Authors:** Zheng Sun, Qiuhang Zhang, Huan Zhao, Chengxi Yan, Hsin-Jung Yang, Debiao Li, Kuncheng Li, Zhi Liu, Qi Yang, Rohan Dharmakumar

**Affiliations:** 1 Department of Radiology, Xuanwu Hospital, Capital Medical University, 100053 Beijing, China; 2 Biomedical Imaging Research Institute, Cedars Sinai Medical Center, Los Angeles, CA 90048 USA; 3 Department of Cardiology, Xuanwu Hospital, Capital Medical University, Beijing, 100053 China; 4 Department of Medicine, University of California in Los Angeles, Los Angeles, CA 90095 USA

**Keywords:** Acute myocardial infarction, Area‐at‐risk, Cardiovascular magnetic resonance, CE-SSFP, SPECT

## Abstract

**Background:**

Contrast-enhanced (CE) steady-state free precession (SSFP) CMR at 1.5T has been shown to be a valuable alternative to T2-based methods for the detection and quantifications of area-at-risk (AAR) in acute myocardial infarction (AMI) patients. However, CE-SSFP’s capacity for assessment of AAR at 3T has not been investigated. We examined the clinical utility of CE-SSFP and T2-STIR for the retrospective assessment of AAR at 3T with single-photon-emission-computed tomography (SPECT) validation.

**Materials and methods:**

A total of 60 AMI patients (ST-elevation AMI, n = 44;  non-ST-elevation AMI, n = 16) were recruited into the CMR study between 3 and 7 days post revascularization. All patients underwent T2-STIR, CE-bSSFP and late-gadolinium-enhancement CMR. For validation, SPECT images were acquired in a subgroup of patients (n = 30).

**Results:**

In 53 of 60 patients (88 %), T2-STIR was of diagnostic quality compared with 54 of 60 (90 %) with CE-SSFP. In a head-to-head per-slice comparison (n = 365), there was no difference in AAR quantified using T2-STIR and CE-SSFP (R^2^ = 0.92, *p* < 0.001; bias:-0.4 ± 0.8 cm^2^, *p* = 0.46). On a per-patient basis, there was good agreement between CE-SSFP (n = 29) and SPECT (R^2^ = 0.86, *p* < 0.001; bias: − 1.3 ± 7.8 %LV, *p* = 0.39) for AAR determination. T2-STIR also showed good agreement with SPECT for AAR measurement (R^2^ = 0.81, *p* < 0.001, bias: 0.5 ± 11.1 %LV, *p* = 0.81). There was also a strong agreement between CE-SSFP and T2-STIR with respect to the assessment of AAR on per-patient analysis (R^2^ = 0.84, *p* < 0.001, bias: − 2.1 ± 10.1 %LV, *p* = 0.31).

**Conclusions:**

At 3T, both CE-SSFP and T2-STIR can retrospectively quantify the at-risk myocardium with high accuracy.

## Background

Myocardial infarct (MI) size assessed on the basis of cardiovascular magnetic resonance (CMR) has been shown to have significant prognostic value [1, 2]. However, emerging evidence supports the notion that infarct size alone may not provide a true representation of the severity of ischemic injury [3, 4]. In the setting of acute myocardial infarction (AMI), the ability to noninvasively differentiate infarcted from at-risk myocardium provides an attractive surrogate endpoint in the assessment of novel therapies to reduce infarct size.

Late gadolinium enhancement (LGE) CMR is the non-invasive reference standard for determining infarct size [5]. When combined with T2-based CMR, it becomes possible to differentiate reversible from irreversible injury and quantify myocardial salvage after coronary revascularization [6]. At-risk myocardium, which is known to have increased free-water content, appears bright on T2-weighted acquisitions, such as triple-inversion turbo spin-echo sequence (T2-STIR) [7, 8]. Several studies have demonstrated that contrast-enhanced (CE) steady-state free precession (SSFP) at 1.5T can also quantify the reversibly injured myocardium [9–12]. An attractive feature of CE-SSFP is that it can be performed in the intervening time period between contrast administration and acquisition of LGE images. This obviates the need for additional T2 acquisitions and reduces the total duration of CMR exams, which is particularly important in AMI patients [13]. Moreover, CE-SSFP has been validated against single photon emission computed tomography (SPECT) in patients for the assessment of at-risk myocardium with relatively high accuracy at 1.5T [12]. To date however, whether the utility of CE-SSFP can be carried over to 3T is not known. Given the growing use of 3T and well-known signal-to-noise ratio (SNR) benefits, which can be traded off for imaging speed or higher-spatial resolution, we investigated the capacity of 3T CE-SSFP against commonly employed T2-STIR for the retrospective determination of area at risk (AAR) in patients with AMI based on SPECT validation.

## Materials and methods

### Study population

The study protocol and procedures were approved by the Ethics Committee of Xuanwu Hospital of Capital Medical University. Written informed consent, in accordance with the Declaration of Helsinki, was obtained from each subject priror to enrollement in the study. The inclusion criteria were: (i) Patients with ST elevation myocardial infarction (STEMI) or non-STEMI (NSTEMI); (ii) age: 18–75 years; and (iii) greater than 0.2 mV in 2 contiguous leads with symptom duration lasting less than 6 hrs. We identified inferior STEMI as an additional ST-segment depression in 2 contiguous anterior leads with a total ST-segment deviation (inferior ST-segment elevation plus anterior ST-segment depression) of ≥ 0.8 mV. Exclusion criteria included: (i) history of severe allergic reaction or hypersensitivity to contrast media; (ii) estimated glomerular filtration rate (eGFR) < 60 ml/min/1.73m^2^; (iii) contraindications to CMR. In addition, patients with cardiac arrest, previous acute MIs, previous percutaneous coronary intervention (PCI) or coronary artery bypass grafting, known heart failure, hepatic failure, recent stroke, coagulopathy, pregnancy, or heart failure (Killip class II to IV) at presentation were also excluded. From Dec 2016 to May 2019, AMI patients (n = 60 (STEMI, n = 44; NSTEMI, n = 16), 59 ± 9 years, 95 % male) were recruited into the study between 3 and 7 days post PCI.

### Coronary angiography

Invasive coronary angiography was used to determine the culprit vessel [14]. All patients were treated with primary PCI with coronary stenting.

### CMR acquisition

CMR was performed on a whole-body 3T CMR scanner (Magnetom Verio; Siemens Healthineers, Erlangen, Germany) with patients positioned in supine position. All images were acquired at end-expiratory position of breath hold with electrocardiograpic (ECG) gating. Scout images were used to localize the heart and the hearts were carefully shimmed. Subsequently, T2-STIR images were acquired in the short-axis view, covering the left ventricle (LV) from the base to the apex with no gap between slices. Approximately 5 minutes after an intravenous injection of 0.2 mmol/kg of gadolinium-based contrast agent (Magnevist, Bayer Healthcare, Berlin, Germany), cardiac phase-resolved CE-SSFP acquisitions that were slice matched to the T2-STIR images were acquired. Fifteen minutes after injection of the contrast agent, inversion-recovery-prepared gradient-echo-based slice-matched LGE images were acquired. Scan parameters for T2-STIR were: repetition time = 2 R-R interals, echo time = 70ms, flip angle = 180°, slice thickness = 6mm, image resolution = 1.3 × 1.3mm^2^; Scan parameters for CE-SSFP were: repetition time = 2.84ms, temporal resolution = 39.2ms, echo time = 1.25ms, flip angle = 50°, slice thickness = 6mm, image resolution = 1.4 × 1.8mm^2^; Scan parameters for LGE were: repetition time = 904ms, echo time = 1.98ms, flip angle = 20°, slice thickness = 6mm, image resolution = 1.4 × 1.9mm^2^.

### CMR image analysis

Imaging slices were anonymized and randomized prior to analysis. T2-STIR and CE-SSFP images were scored between 1 and 3 for image quality by two radiologists blinded to the identity of the images as follows: ‘non-diagnostic’ =1; ‘acceptable’=2; and ‘good’=3. Images deemed ‘non-diagnostic’ were identified as those where myocardium could not be evaluated due to imaging artefacts or poor signal characterisitcs. Images identified as ‘acceptable’ were those where the AAR could be identified, even though some image quality issues were present. Those images identified as ‘good’ were those without any of the issues above. The images identified as ‘good’ or ‘acceptable’ were deemed to be of diagnostic quality. On per-patient analyses, all images were scored as ‘good’ or ‘acceptable’; and ‘non-diagnostic’ cases were where at least one slice was scored as ‘non-diagnostic’ and had to be excluded. Images were analyzed by two observers and disagreements in image quality between the reviewers were resolved in consensus. SNR and contrast-to-noise (CNR) were measured for T2-STIR and CE-SSFP respectively. SNR was calculated as the mean signal intensity within the affected region (at-risk or infarcted myocardium) divided by the standard deviation of signal intensities within a background region of interest in air. CNR was calculated as the difference in SNR between the affected and remote myocardium. Insufficient CNR was defined as a CNR of less than 5 between the myocardium and the blood pool.

#### (i) Qualitative analysis

Two radiologists with greater than 10 years of CMR experience, blinded to the patients’ clinical history, independently evaluated the images. Each reader assessed for the presence or absence of hyperintense zones in each short-axis slice and ascribed it to a segment in accordance with the 16-segment model of the American Heart Association.

#### (ii) Quantitative analysis

Quantitative CMR image analysis was performed using cvi^42^ (version 5.12.1 (1686), Circle Cardiovascular imaging Inc, Calgary, Alberta, Canada). T2-STIR and CE-SSFP images were also randomized and independently analyzed by the two blinded reviewers. T2-STIR and CE-SSFP images were analyzed as follows: (i) endocardial and epicardial borders of the LV were traced in all short-axis images to segment the myocardium; and (ii) placing a region of interest (ROI) in the remote myocardium not affected by infarction to determine the hyperintense region using the mean + 2 standard deviation (SD) criterion, as previously described [11]. The papillary muscles were excluded in the analysis. On per-patient analysis, AAR was computed from the sum of the area of hyperintense myocardium from all short-axis slices multiplied by the slice thickness and normalized to the volume of LV myocardium and reported as percentage of LV. Areas of hypointense core (i.e., microvascular obstruction/intramyocardial hemorrhage) were manually included as part of the AAR [15]. Infarcted myocardium was determined following, endocardiand and epicardial contouring to delineate the myocardium in LGE images, and then applying the mean + 5SD crierion to determine the area of infarction [16]. In this thresholding process, remote myocardium was defined as the area diametrically opposite to the hyperintense zone of myocardium in LGE images.

### SPECT acquisition and analysis

Thirty patients (all presenting with STEMI) underwent SPECT. Prior to PCI, patients received a body weight-adjusted injection of sestamibi (MIBI (350-700MBq, i.v., Cardio-lite, Lantheus Medical Imaging, North Billerica, Massachusetts, USA). Post PCI, patients were placed in supine position and imaged at 5.6^o^ increments (64 × 64 matrix, with isotropic resolution of 6.8 mm×6.8 mm×6.8 mm). SPECT images were analyzed using Myovation (version Xeleris 3, General Electric Healthcare Milwaukee, Wisconsin, USA). The tomographic reconstruction was accomplished using filtered back projection with the Butterworth Filter. The reconstructed data were re-oriented according to the three anatomical axes of the heart. In order to avoid bias, manual intervention was performed only when the automatic LV alignment was incorrect. An automatic segmentation algorithm was used to find the centerline through the LV wall to identify the endo- and epi-cardium based on an individually estimated wall thickness and signal intensity values within the image. Manual adjustments of the automatic delineations were sometimes required in the LV outflow region. Subsequently, seventeen-segment polar maps were constructed and tracer uptake information for each segment was determined for all patients. Myocardial perfusion defect on SPECT images were used to delineate the AAR as previously described [17, 18].

### Statistical analysis

Statistical analysis was performed using SPSS (version 19.0, Statistical Package for the Social Sciences, International Business Machines, Inc., Armonk, New York, USA). Continuous variables were expressed as mean ± standard deviation, and categoric data are presented as absolute numbers and percentages. Statistical significance was set at *p* < 0.05. A paired t-test was used to compare the means of the SNR, CNR of the AAR and remote myocardium from T2-STIR and CE-SSFP images, as well as the difference in AAR determined using T2-STIR and CE-SSFP images. For continuous variables, agreement in AAR determined using T2-STIR and CE-SSFP images and interobserver variability was assessed using Bland-Altman analysis. Inter-observer agreement in AAR determined using T2-STIR and CE-SSFP images was evaluated using one-way random intraclass correlation coefficient (ICC).

## Results

### Study population

**Fig. 1:**
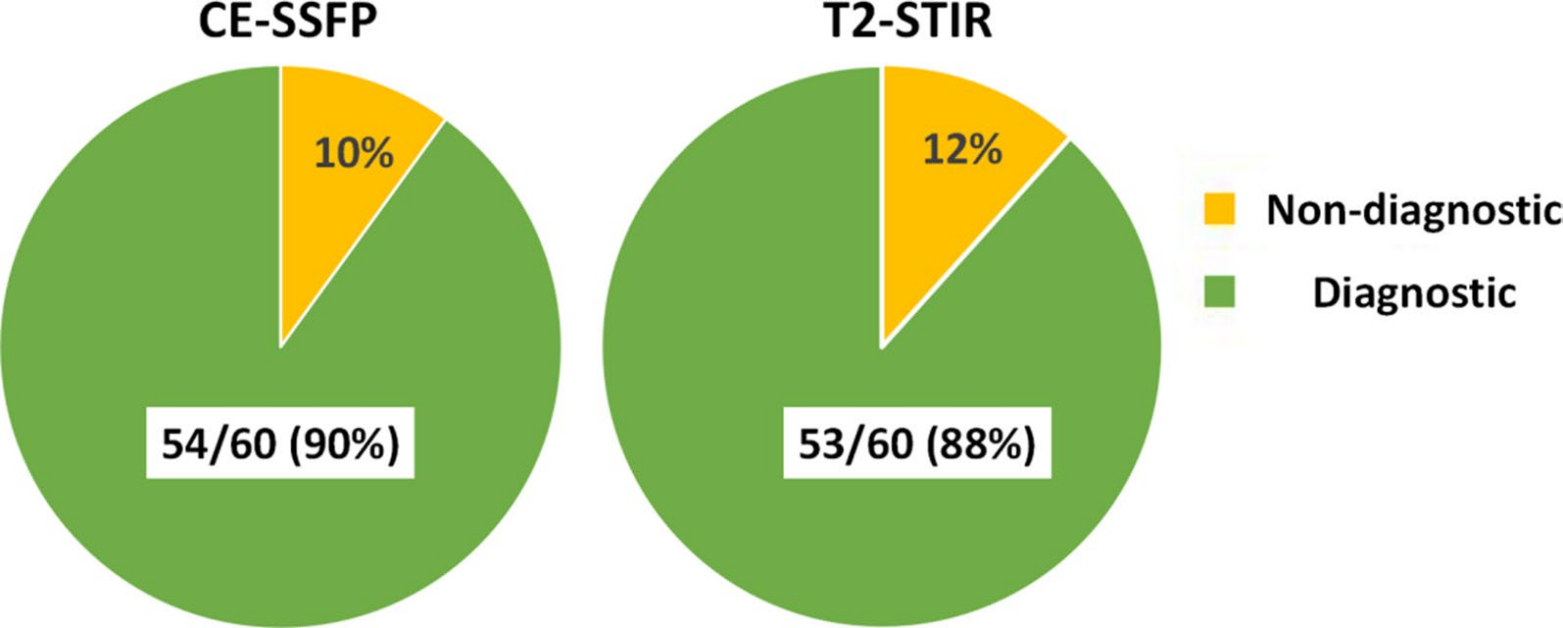
Breakdown of Diagnostic and Non-diagnostic Cases. Diagnostic refers to cases where all images were scored as ‘good’ or ‘adequate’. *Non-diagnostic* refers to cases where at least one imaging slice was scored as non-diagnostic and had to be excluded from per-patient analysis. Criteria for ‘good’, ‘adequate’ and ‘non-diagnostic’ scores are defined in the text

Out of 60 patients, 53 patients (88 %) had diagnostic T2-STIR images and 54 patients (90 %) had diagnostic CE-SSFP images (Fig. 1). T2-STIR data from seven patients were excluded due to incomplete suppression of blood pool (n = 1), poor SNR (n = 1), and motion artifacts (n = 5). CE-SSFP data from six patients were excluded due to banding artifacts (n = 3) or insufficient CNR (n = 3). Collectively there were 50 patients with matched T2-STIR and CE-SSFP images for joint evaluation.

**Fig. 2:**
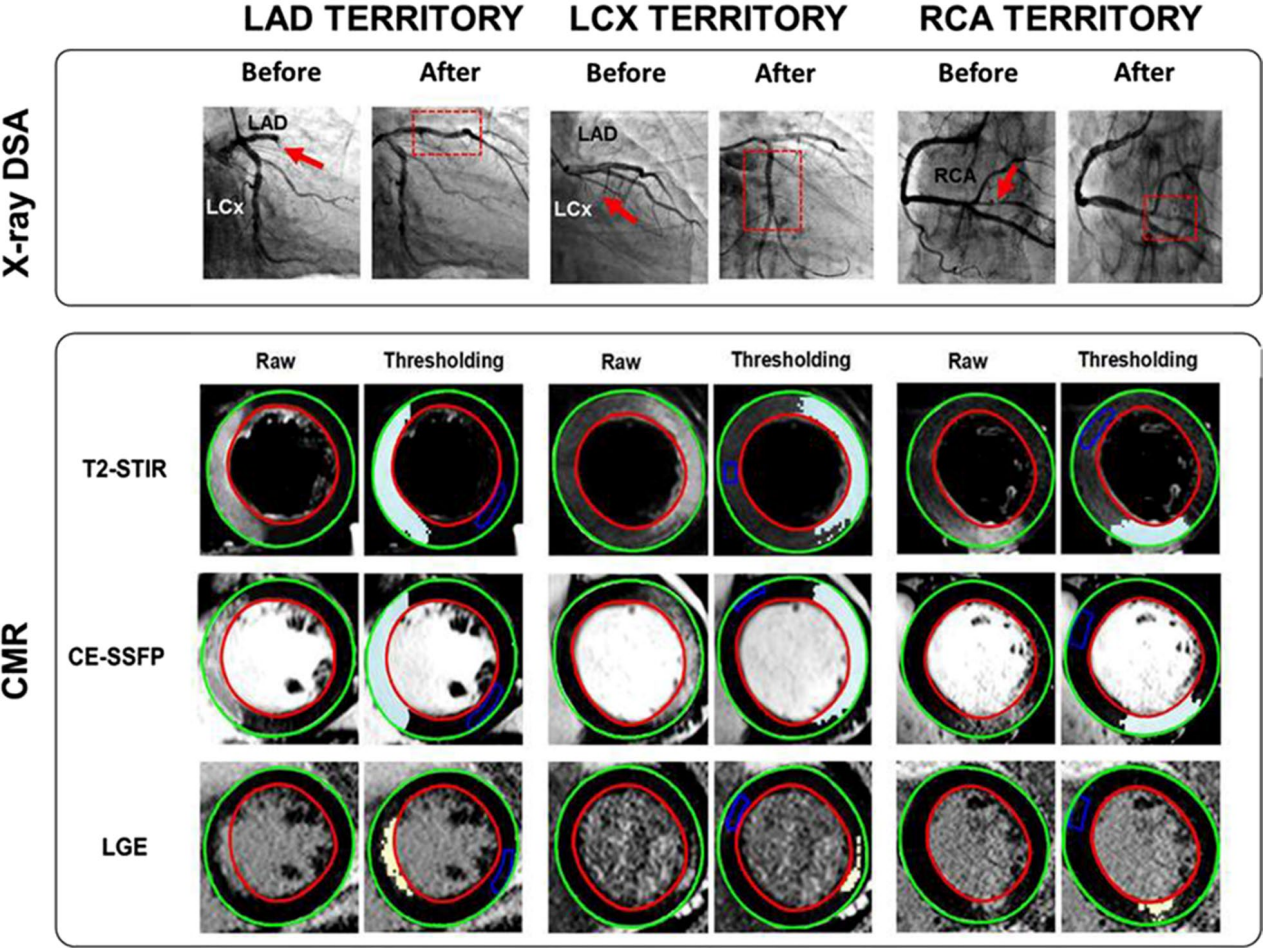
Representative Images from non-ST elevation myocardial infarction (NSTEMI) Patients with left anterior descending (LAD), left circumflex (LCx) and right coronary artery (RCA) myocardial infarctions. X-ray Digital subtraction angiography (DSA) images acquired before and after PCI, along with corresponding short-axis myocardial T2-STIR and contrast enhanced (CE) steady state free precession (SSFP) showing at-risk regions and infarct territories (late gadolinium enhancement (LGE)) acquired at 3T are shown. On X-ray DSA images, the red arrows refer to occlusive or substantially narrowed vessels, and the red box with dotted line demonstrates the re-establishment of blood flow following recanalization of the corresponding vessel. On CMR images, epicardial and endocardial borders are delineated by green and red borders, respectively. The left columns shows the raw images and the right columns shows the same images processed using semi-automatic threshold-based signal detection. The edematous myocardium delineated by the T2-STIR and CE-SSFP images are shown as a region enclosed by the pale blue areas and irreversibly damaged myocardium is identified on LGE images as yellow areas

**Fig. 3:**
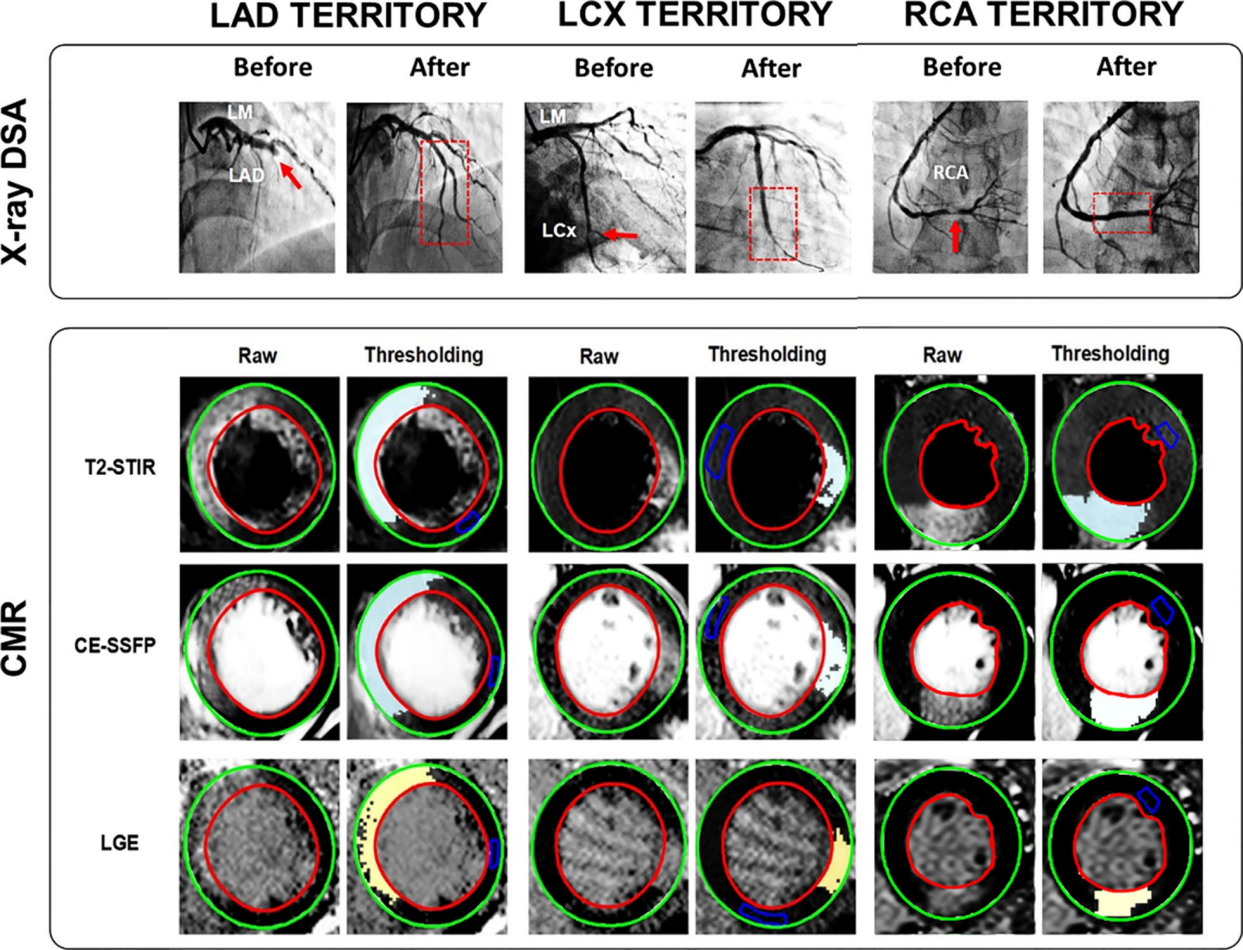
Representative Images from STEMI Patients with LAD, LCx and RCA Myocardial Infarctions. X-ray Digital subtraction angiography (X-ray DSA) images acquired before and after PCI, along with corresponding short-axis myocardial T2-STIR and CE-SSFP showing at-risk regions and infarct territories (LGE) acquired at 3T are shown. On X-ray DSA images, the red arrows refer to occlusive or substantially narrowed vessels, and the red box with dotted line demonstrates the re-establishment of blood flow following recanalization of the corresponding vessel. On CMR images, epicardial and endocardial borders are delineated by green and red borders, respectively. The left columns shows the raw images and the right columns shows the same images processed using semi-automatic threshold-based signal detection. The edematous myocardium delineated by the T2-STIR and CE-SSFP images are shown as a region enclosed by the pale blue areas and irreversibly damaged myocardium is identified on LGE images as yellow areas

Culprit coronary arteries determined on the basis of invasive coronary angiography were as follows: left anterior descending coronary artery (LAD) (n = 30, 50 %), right coronary artery (RCA) (n = 18, 30 %) and left circumflex coronary artery (LCx) (n = 12, 20 %). Baseline characteristics between STEMI and NSTEMI patients were similar (all p < 0.05). Detailed demographics and clinical characteristics of the patients enrolled in this study are provided in Table 1. Figures 2 and 3 show representative examples in STEMI and NSTEMI patients with AAR delineated from T2-STIR and CE-SSFP, as well as MI regions in LGE images, along with pre- and post-PCI X-ray digital-subtraction-angiographic (DSA) images.

**Table 1:** Characteristics of acute myocardial infarction (MI) patients

Characteristics	STEMI (n = 44)	NSTEMI (n = 16)	*p*-value
Age (yrs)	58 ± 13	61 ± 7	0.40
Male/female/(Male gender %)	42/2/ (95)	15/1/ (94)	0.57
Height (cm)	172 ± 7	171 ± 6	0.67
Weight (kg)	74 ± 9	75 ± 9	0.91
Body mass index	25 ± 3	26 ± 3	0.78
LV end diastolic volume (mL)	89 ± 35	85 ± 35	0.92
LV end systolic volume (mL)	43 ± 25	39 ± 19	0.23
LV ejection fraction (%,median,IQR)	52 (41–59)	50 (40–60)	0.50
TIMI flow pre-PCI 0–1 /n (%)	30 (68)	4 (25)	0.89
TIMI flow post-PCI 2–3 /n (%)	14 (32)	12 (75)	0.32
Hypertension/n (%)	24/44 (55)	8/16 (50)	–
Diabetes/n (%)	10/44 (23)	6/16 (38)	–
Smoker/n (%)	29/44 (66)	14/16 (88)	–
Culprit vessel by angiography			
LAD (n)	22	8	–
LCx (n)	9	3	–
RCA (n)	13	5	–

Values are mean ± SD or n (%)

LAD, left anterior descending coronary artery; LCx, left circumflex coronary artery; LV, left ventricular; RCA, right coronary artery; NSTEMI, non-ST elevation myocardial infarction; PCI, percutaneous coronary intervention; STEMI, ST elevation myocardial infarction; TIMI, thrombolysis in myocardial infarction

### Image quality: T2-STIR vs. CE-SSFP at 3T

**Fig. 4:**
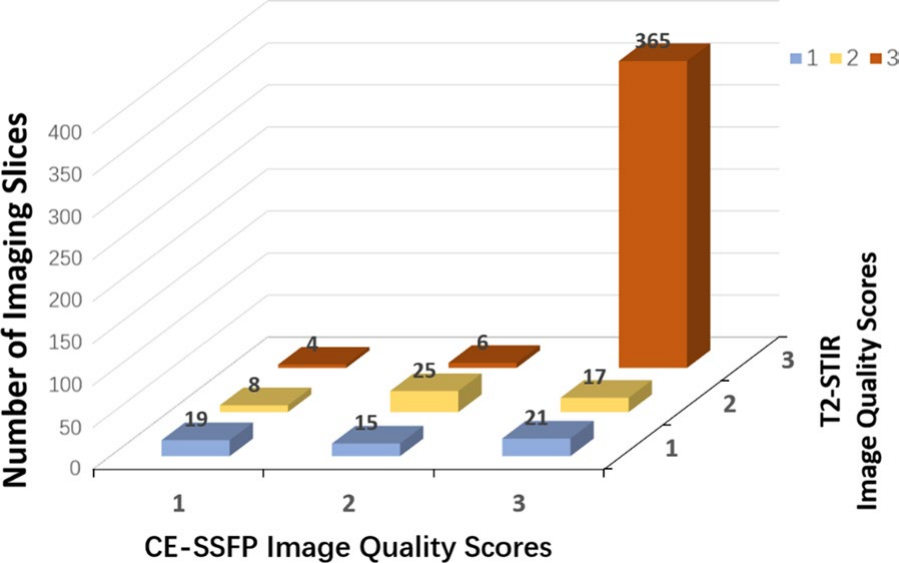
Image Quality: T2-STIR versus CE-SSFP. Diagnostic scores of T2-STIR and CE-SSFP are shown (score are as follows: ‘non-diagnostic’ is scored 1; ‘adequate’ scored 2; and ‘good’ scored 3). In total, 365 matched imaging slices from T2-STIR and CE-SSFP had the highest image quality scores and were used for head-to-head comparison

Image quality was deemed to be ‘good’ in 78.1 % (375/480) and ‘acceptable’ in 10.4 % (n = 50) in T2-STIR images. In comparison, 84.0 % (403/480) of the images were deemed ‘good’ and 9.6 % (n = 46) were ‘acceptable’ with CE-SSFP. One hundred and five T2-STIR imaging slices were excluded due to incomplete suppression of blood pool (n = 45), low SNR (n = 28), or motion artifacts (n = 32). Seventy seven CE-SSFP imaging slices were excluded due to banding artifacts (n = 41) or insufficient CNR (n = 36). Once slice matched, a total of 365 pairs of T2-STIR and CE-SSFP images were deemed to have ‘good’ image quality for head-to-head comparison. For additional details see Fig. 4. Collective image quality scores were: 2.6 ± 0.7 (T2-STIR) vs 2.8 ± 1.0 (CE-SSFP), *p =* 0.021.

### SNR and CNR: T2-STIR vs. CE-SSFP at 3T

On per-slice analysis, there were 144 matched imaging slices with ‘good’ image quality with evidence of hyperenhancement in T2-STIR and CE-SSFP images. In the affected area, the SNR of CE-SSFP images was significantly higher than the SNR of T2-STIR images (83 ± 67 vs. 51 ± 38, *p =* 0.0024). CNR between the affected and remote regions was also significantly higher in CE-SSFP images compared to T2-STIR images (31 ± 31 vs. 16 ± 13, *p =* 0.0108).

### Edema based detection of AAR: T2-STIR vs. CE-SSFP images at 3T

**Fig. 5:**
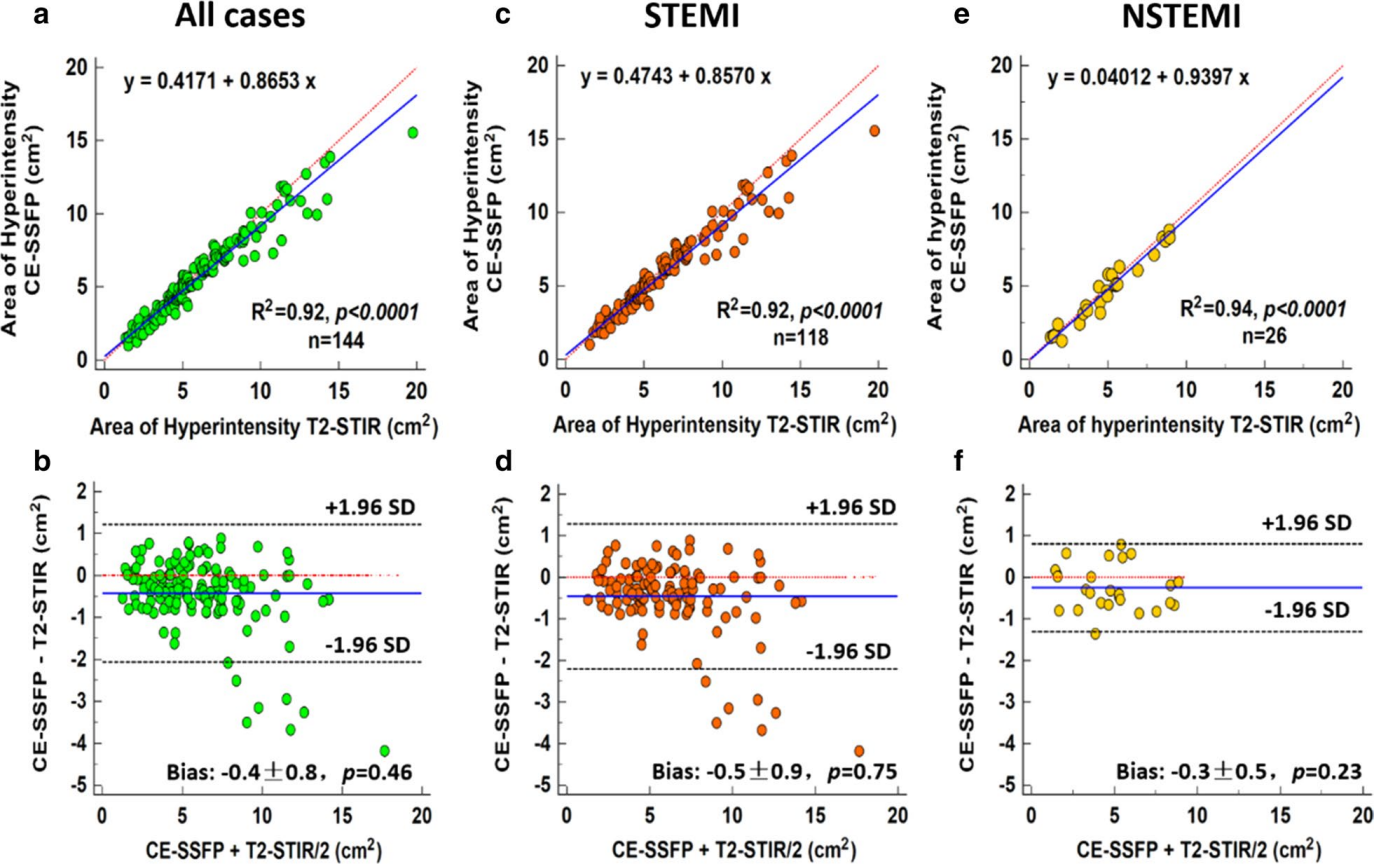
Comparison of T2-STIR and CE-SSFP AAR in Per-Slice Analysis*.* Linear regression between area at risk (AAR) determined using diagnostic quality T2-STIR and CE-SSFP images (blue line showing line of best fit; red line represents line of identity) for both STEMI and NSTEMI patients are shown in (A) and the corresponding Bland-Altman analysis is shown in (B). Similar regression and Bland-Altman plots are shown for STEMI ((C) and (D)) and NSTEMI (E) and (F)). In all cases, the AAR is expressed as area of hyperintensity in the imaging slice

AAR determined collectively from STEMI and NSTEMI patients using T2-STIR and CE-SSFP showed excellent agreement (R^2^ = 0.92; *p* < 0.001; bias =-0.4 ± 0.8cm^2^, *p* = 0.46) (Fig. 5). The agreement in AAR between T2-STIR and CE-SSFP was also excellent in the individual groups: STEMI group (R^2^ = 0.92; *p* < 0.001; bias=-0.5 ± 0.9 cm^2^, *p* = 0.75); and NSTEMI group (R^2^ = 0.94; *p* < 0.001; bias=-0.3 ± 0.5cm^2^, *p* = 0.23). There was no significant difference in inter-observer agreement in the assessment of AAR with either of the methods (Observer 1: T2-STIR vs. CE-SSFP, *p =* 0.683; Observer 2: T2-STIR vs. CE-SSFP, *p =* 0.552; ICC > 0.94 for both T2-STIR and CE-SSFP; for additionals details see Table 2).

**Table 2:** Inter-observer agreement for Edema-based at-risk myocardium area on T2-STIR and CE-SSFP images

	T2-STIR	CE-SSFP	*p*-value
Observer 1 (cm^2^)	5.84 ± 2.13	5.45 ± 2.39	0.683
Observer 2 (cm^2^)	5.77 ± 2.26	5.63 ± 2.25	0.552
ICC*	0.96 [0.94–0.97]	0.94 [0.91–0.96]	–

* Data are percentages, with raw data in parentheses and 95 % confidence intervals in brackets

### Validation: CE-SSFP and T2-STIR vs. SPECT

**Fig. 6:**
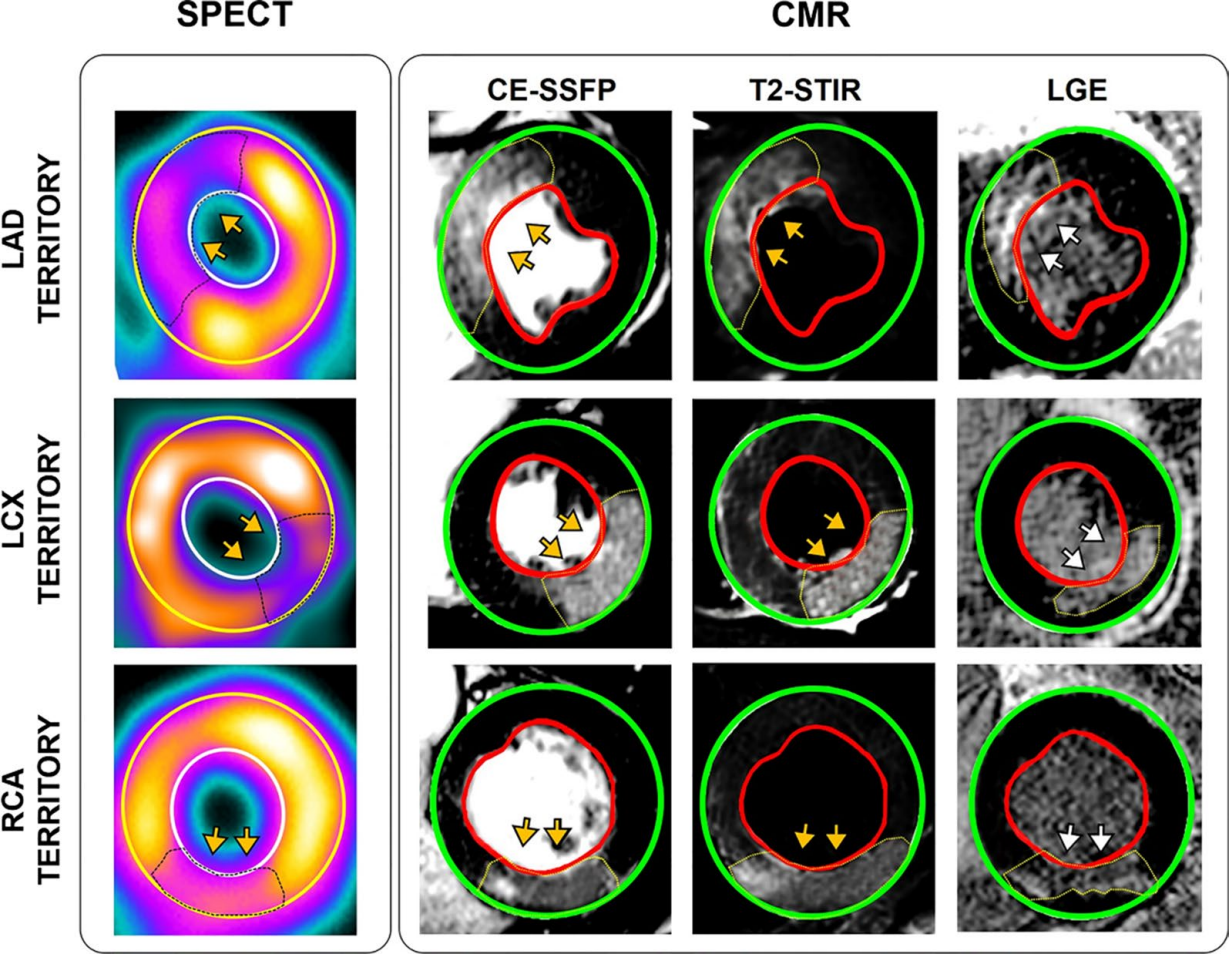
SPECT Validation in Acute MI Patient with LAD, LCx and RCA Myocardial Infarctions. The AAR determined using SPECT, CE-SSFP, T2-STIR, as well as infarct size determined using LGE images are shown from representative LAD, LCx and RCA myocardial infarctions. Yellow arrows demarcate the at-risk myocardium and the white arrows point to the infarct zone

Representative examples of AAR in the left anterior descending (LAD), left circumflex (LCx) and right coronary artery (RCA) territories identified using SPECT, along with closely matched slices of CE-SSFP, T2-STIR images, and LGE are shown in Fig. 6.

**Fig. 7:**
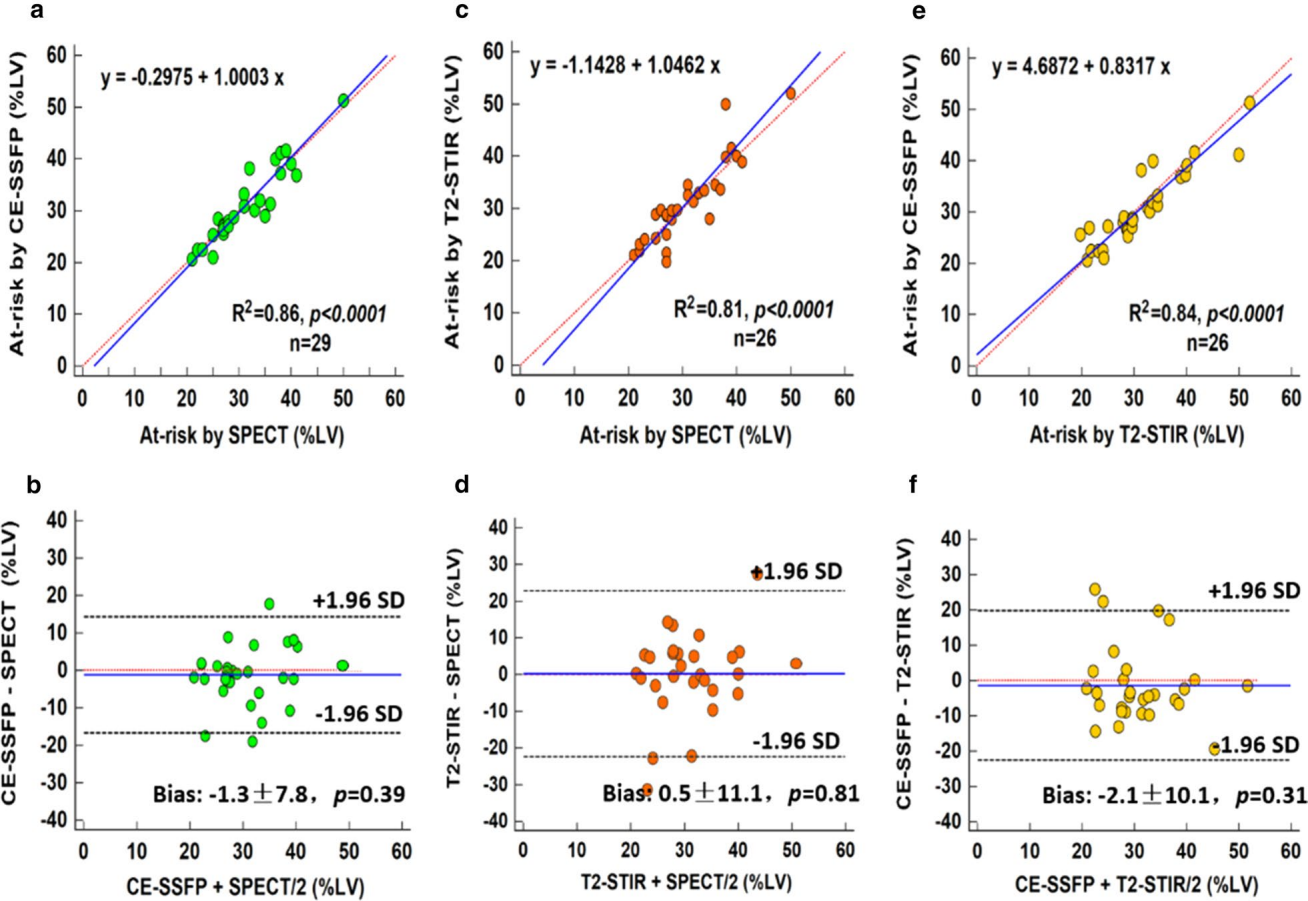
AAR from SPECT vs. AAR from T2-STIR and CE-SSFP*. ***a** There was good agreement between the CE-SSFP (mean 30 ± 7 %LV) and SPECT (mean 28 ± 7 %LV) with an R^2^ of 0.86; (*p* < 0.001) with a low bias on Bland Altman analysis (mean ± 2SD: bias − 1.3 ± 7.8 %LV, *p* = 0.39) as seen on **b**. **c**, **d** T2-STIR (mean 31 ± 11 %) vs. SPECT on per patient-based analysis showed good correlation (R^2^ of 0.81; *p* < 0.001) and a mean difference of 0.5 ± 11.1 %LV; *p* = 0.81). **e**, **f** A strong agreement between CE-SSFP and T2-STIR was observed (R^2^ = 0.84; *p* < 0.001) with a low bias (− 2.1 ± 10.3 %LV; *p* = 0.31)

The range in AAR from SPECT was 21 to 50 % (mean 28 ± 7 %) of the LV; and from CE-SSFP was 20 to 51 % (mean 30 ± 7 %) of the LV. As illustrated in Fig. 7A there was a strong correlation between AAR determined from CE-SSFP and SPECT (R^2^ = 0.86, *p* < 0.001). The difference between the enhanced region on CE-SSFP and on SPECT was − 1.3 ± 7.8 %LV, *p* = 0.39; see Fig. 7B. T2-STIR also showed good agreement with SPECT for AAR measurement (R^2^ = 0.81, *p* < 0.001; bias: 0.5 ± 11.1 %LV, *p* = 0.81). The relative performance in AAR on a per-patient basis with T2-STIR and CE-SSFP are shown in Fig. 7E and F. Notably, there was also a strong agreement between CE-SSFP and T2-STIR with respect to the assessment of AAR on per-patient analysis (R^2^ = 0.84, *p* < 0.001, bias: − 2.1 ± 10.1 %LV, *p* = 0.31).

## Discussion

To our knowledge, this is the first study to evaluate the capability of CE-SSFP and T2-STIR for retrospective determinations of the at-risk myocardium at 3T with SPECT validation [12, 19]. We found that CE-SSFP at 3T can identify the the AAR equally well as T2-STIR. In the current study, T2-STIR images were diagnostic in 53 of 60 patients (88 %) and CE-SSFP images were diagnostic in 54 of 60 patients (90 %) for determining AAR on a per-patient basis. When comparing our findings here to the larger multi-vendor, multi-center study at 1.5T, which was performed in 215 AMI patients, only 65 % of the subjects had diagnostic T2-STIR images compared to 97 % with CE-SSFP [20]. Thus it appears that on a per-patient basis, albeit from a smaller group of AMI patients, the fraction of diagnostic T2-STIR images were greater, while the fraction of diagnostics CE-SSFP images were slightly lower. This suggests that T2-STIR may perform better at 3T than at 1.5T. However, our findings here are limited to a single CMR vendor sytem at 3T. Additional studies are needed to confirm whether our observations would extend to other vendors and systems as well since in a multi-vendor study at 1.5T [20], there were marked differences in the fraction of T2-STIR images with diagnostic image quality across vendors, which was not the case with CE-SSFP.

Further, in our study, the AAR determined using T2-STIR and CE-SSFP showed good agreement (R^2^ = 0.84, p < 0.001 and bias of − 2.1 ± 10.1 %, p = 0.31). This is consistent with the previous multi-center, multi-vendor study [20], which also showed good agreement between T2-STIR and CE-SSFP based assessment of AAR (R^2^ = 0.71, p < 0.001 and bias of 0.02 ± 6 %). Thus our findings here show that when images are of diagnostic quality, both T2-STIR and CE-SSFP can both accurately quantify AAR with near-perfect agreement at 1.5T and 3T. Notwithstanding our favorable findings here, additional studies are required to establish whether our findings can hold across other scanner platforms and patient demographics.

In spite our observations and those of others [9–11, 21], all collectively favoring the notion that edema based CMR approaches can enable quantification of AAR, whether CMR can provide restropective assessment of AAR remains controversial [20]. This is highlighted in an expert-opinion paper, where only a non-unanimous support was reached to not recommend the use of edema-based imaging of AAR [22]. Our data here, at least on the basis of SPECT validation, supports the notion that AAR determined on the basis of zone of hyperintensity in CE-SSFP and T2-STIR (likely from edema) are equivalent. While our results lend support to the idea that edema may be used as a marker of AAR, whether the CE-SSFP and T2-STIR approaches at 3.0T indeed are met with stable edema signals across physiological conditions (e.g., one that is not altered in response to therapy) to identify the true AAR remains to be investigated. Should AAR based on edema imaging at 3T prove to be valuable, a combined evaluation of both LGE and CE-SSFP would enable differentiating the type of myocardial injury (reversible vs. irreversible) and delineation of salvageable myocardium in response to ischemia [23, 24]. As suggested by previous studies, the capacity to ascertain the extent of myocardial salvage on the basis of CMR would be immensely valuable in the evaluation of novel therapies aimed at reducing infarct size [2, 4, 25]. From a technical standpoint, 3.0T based determination of AAR with CE-SSFP imaging may offer additional opportunities, most notably trading off the higher SNR at 3T for increased imaging speed or spatial resolution. However, these potential technical advantages at 3T would need to be evaluated within the constraints of excellent CE-SSFP image quality that is already available at at 1.5T.

Although T2-STIR has been shown to be promising in the assessment of AAR at 1.5T, double-inversion recovery magnetization preparation is known to impose certain limitations, such as difficulties in distinguishing blood pool from myocardium, particularly in the apical parts of the LV where hypokinesia and trabeculation result in stagnant blood flow [26, 27]. This problem can be further accentuated by B1 inhomogeneities at 3T, which can also result in incomplete suppression of blood pool compared to 1.5T [28]. CE-SSFP was initially introduced as an alternative for overcoming these difficulties at 1.5T. Given the potentially increased B0 and B1 inhomogeneities at 3T, which may hamper T2-STIR more significantly than CE-SSFP, our findings on SNR and CNR differences observed in CE-SSFP compared to T2-STIR approach are not surprising. However, when comparing the differences in the fraction of diagnostic image quality, we found that the per-slice T2-STIR images quality was poorer than per-slice CE-SSFP (22 % (T2-STIR) vs. 16 % (CE-SSFP)). However, the loss due to lack of diagnostic image quality in T2-STIR and CE-SSFP on per-patient basis were nearly the same (12 % (T2-STIR) vs. 10 % (CE-SSFP)), suggesting that both T2-STIR and CE-SSFP offer comparable diagnostic images at 3T.

The use of CE-SSFP at 3T however needs additional care since the heart has to be shimmed carefully to avoid banding artifacts, which are more common at 3T than 1.5T in balanced SSFP acquisitions. The ~ 10 % of the CE-SSFP imaging slices that were not included in the analysis were due to banding artifacts or poor SNR, likely stemming from the well-known off-resonance artifacts in SSFP and potential B1 inhomogeneities contributing to loss of SNR at 3T. Further, at 1.5T, the agreement between CE-SSFP and SPECT for AAR was good (R^2^ = 0.78 with a bias of 0.5 %) [12], which also compares favorably with our data at 3T (R^2^ = 0.86, bias of 1.3 %). Thus on the balance, based on our findings here at 3T and the reports in the literature at 1.5T, it appears that both 1.5T and 3T can be used interchangeably to assess AAR with CE-SSFP.

We analysed both the CE-SSFP and T2-STIR images using manual contouring, which is time consuimg and is not ideal in the clinical setting. Approaches that can automate contouring of CE-SSFP and T2-STIR images for the purpose of quantifying at-risk myocardium have been developed [29]. While these approaches have been shown to have excellent performance against manual contouring on 1.5T images, they need to be evaluated on CE-SSFP and T2-STIR images at 3T. If validated, these automatic segmentation approaches could markedly improve the analysis and quantification of AAR and thus further enhance the utility of the approaches in the clinical setting that employ CMR at 3T.

Finally, a key finding from our study is that the AAR determined based on CE-SSFP CMR at 3T and established reference standard, SPECT, are highly correlated and have minimal/negligible bias. Nothwithstanding this, our findings here also provide the first clinical support for using T2-STIR for ascertaining the AAR at 3T. Based on this finding we anticipate that other T2-based approaches, including T2 mapping, are also likely to perform at least as well, but further studies are needed to confirm this.

## Limitations

There were a few limitations with the current study. First, the limitations of SPECT for estimation of AAR, particularly spatial resolution, should be recognized. However, SPECT is an established independent reference standard for determining AAR. Next, the present study was performed in a small number of reperfused AMI patients. As such, the findings here should be taken as the first evidence in support of CE-SSFP for retrospective assessment of at-risk myocardium at 3T CMR. In addition, as indicated earlier, the true capability of the CE-SSFP and T2-STIR at 3T for quantifying the AAR remains to be tested in therapeutic settings to assess whether edema signals can remain unaltered by the therapies of interest. Additional studies may be needed to further evaluate potential variability with respect to coronary territory. Finally, studies across a broader spectrum of AMI patients and 3T scanner systems are needed.

## Conclusions

At 3T, both CE-SSFP and T2-STIR can retrospectively quantify the at-risk myocardium with high accuracy as compared with SPECT.

## Data Availability

The datasets used and/or analysed during the current study are available from the corresponding author on reasonable request.

## Abbreviations

AAR: Area at risk
AMI: Acute myocardial infarction
CE: Contrast-enhanced
CMR: Cardiovascular magnetic resonance
CNR: Contrast-to-noise ratio
DSA: Digital subtraction angiography
ECG: Electrocardiogram
eGFR: Estimated glomerular filtration rate
LAD: Left anterior descending coronary artery
LCx: Left circumflex coronary artery
LGE: Late gadolinium enhancement
LV: Left ventricle/left ventricular
MI: Myocardial infarction
MVO: Microvascular obstruction
NSTEMI: Non-ST elevation myocardial infarction
PCI: Percutaneous coronary intervention
RCA: Right coronary artery
ROI: Region of interest
SD: Standard deviation
SNR: Signal-to-noise ratio
SPECT: Single photon emission computed tomography
STEMI: ST elevation myocardial infarction
